# Online support seeking, co-rumination, and mental health in adolescent girls

**DOI:** 10.3389/fpsyt.2023.1040636

**Published:** 2023-03-07

**Authors:** Erin Mackenzie, Anne McMaugh, Penny Van Bergen, Roberto H. Parada

**Affiliations:** ^1^Centre for Educational Research, Western Sydney University, Penrith, NSW, Australia; ^2^Macquarie School of Education, Macquarie University, Sydney, NSW, Australia; ^3^School of Education, University of Wollongong, Wollongong, NSW, Australia

**Keywords:** online support seeking, co-rumination, depression, adolescents, anxiety

## Abstract

**Introduction:**

Adolescents frequently use informal support seeking to cope with stress and worries. Past research in face-to-face contexts has shown that the relationship between informal support seeking and mental health is influenced by the specific strategy used and the mode through which support is sought. To date, little research has considered the relationship between support seeking online and adolescent mental health.

**Methods:**

In this study, structural equation modeling (SEM) examined the mediating role of co-rumination in the relationships between seeking support from friends or online and two measures of mental health: depression and anxiety. Participants were 186 adolescent girls, drawn from four independent girls’ schools in Sydney, Australia. Four brief vignettes described common social stressors and participants rated their likelihood of seeking support from close friends and from informal online sources. Co-rumination was measured using a short form of the Co-rumination Questionnaire and depression and anxiety were measured with the youth version of the Depression, Anxiety, and Stress Scale-Youth (DASS-Y).

**Results:**

Different patterns of findings were found for support seeking from close friends and support seeking online. First, support seeking from friends was related to lower levels of depression and anxiety while seeking support online was related to higher levels depression and anxiety. Second, co-rumination suppressed the relationship between seeking support from friends and depression, but not online support seeking and depression or anxiety.

**Discussion:**

These findings suggest that co-rumination reduces the benefits of seeking support from friends but is unrelated to online support seeking. The findings also confirm the problematic nature of online support seeking for adolescent girls’ mental health, particularly in response to social stressors.

## Introduction

Online communication provides adolescents with almost constant access to their friends and peers, potentially allowing interpersonal coping strategies to be more accessible than ever before. Although support seeking from friends is typically viewed as an adaptive coping strategy (1), there is mounting evidence to suggest that informal online support seeking is related to poorer mental health (2–4). Broader evidence suggests that support seeking is likely to be maladaptive if it includes co-rumination: the repeated discussion of problems and associated negative affect with close friends (5, 6). Co-rumination is particularly common in adolescent girls’ friendships and, while it can contribute to stronger friendships (7, 8), it also predicts poorer mental health (9, 10). It is unknown, however, whether co-rumination influences the relationship between traditional or online support-seeking tendencies and mental health.

Support seeking is a common interpersonal coping strategy that is generally considered adaptive, enabling the support seeker to access social resources that can help to deal with stress (11). Starting from the early adolescent period, it is developmentally normative for adolescents to become more reliant on friends for social support and less reliant on their family (12). While support seeking is considered adaptive, the relationship between support seeking and indicators of mental health in adolescents is inconsistent. This is most clearly demonstrated by the results of three meta-analyses, which have attempted to determine the relationship between support seeking and psychosocial outcomes in children and adolescents (12–14). While Chu et al. (13) reported a weak association between support seeking and wellbeing outcomes, Heerde and Hemphill (12) and Compas et al. (14) found that support seeking was not related to internalizing behaviors. In contrast, when examining findings from a small number of longitudinal studies, Compas et al. (14) found that support seeking was related to higher levels of internalizing symptoms.

These equivocal findings suggest that the way in which adolescents seek support from friends may influence the efficacy of support seeking as a coping strategy (8). For example, Vélez et al. (15) found that support seeking was related to lower levels of depression and anxiety in early adolescence, however, this relationship became weaker (and reversed, in the case of depression) for individuals who engaged in high levels of rumination. One possible explanation for these findings is that adolescents who ruminate may be more likely to co-ruminate with their friends while seeking support, which in turn could negate the positive mental health benefits expected from support seeking. This suggests that co-rumination may be an important mediator of the benefits of support seeking and this hypothesis is worthy of investigation.

Reflecting adolescents’ ubiquitous use of digital devices to communicate with friends, research has turned to focus on relationships between seeking support online and mental health. This smaller body of research has reported more consistent findings: that seeking support online represents a less adaptive way of coping with stress for adolescents and is related to increased depressed mood (3), anxiety (4), worry, and loneliness (2). Explanations for this include poorer quality support provision due to reduced social cues (16–18), overly negative or critical feedback in response to support seeking attempts (19), and increased availability of friends resulting in problematic support seeking behaviors such as co-rumination (20). Given the potential for poorer quality support online, any investigation of co-rumination as a potential mediator of the benefits of support seeking should seek to disentangle every day and online support seeking from friends.

While support seeking is commonly associated with beneficial mental health outcomes, co-rumination is often associated with poorer mental health outcomes. Co-rumination is defined as the tendency to excessively talk about and revisit a problem over and over again; often dwelling on the negative emotions and encouraging further problem talk, but not problem solving, with a partner, such as a close friend (21, 22). This behavior has been linked both directly and indirectly to poor adjustment with internalizing symptoms, including depression, and anxiety, reported by adults and adolescents (9, 22). Longitudinal research shows evidence that co-rumination predicts later depression *via* brooding rumination, but depression does not predict later co-rumination (22). However, other studies suggest that the relationship between co-rumination and internalizing symptoms is bidirectional (7, 10). Paradoxically, co-rumination may also have positive trade-offs, with increased friendship quality and closer friendships associated with adolescents’ co-rumination scores (23). Co-rumination scores are typically higher in girls (23) while higher scores are also associated with increased risk of depressive disorder (24, 25). The presence of a friend as the ruminating partner likely explains the positive trade off of high-quality close friendships (21) while the tendency to dwell on problems in these co-ruminating partnerships has been linked to internalizing problems (23).

Perhaps not surprisingly, co-rumination readily occurs in support-seeking contexts (26). Perceived stressors cause individuals to reach out to others for support in managing their emotions, with specific conversational characteristics determining whether the discussion includes more productive forms of shared problem solving and emotional support or a repetitive and non-productive dwelling on the challenge and on the emotions it evokes [see (27)]. Indeed, Stone and Veksler [(9), p. 3] describe how co-rumination might “masquerade as beneficial social support.” While findings are limited, recent research highlights how co-ruminative tendencies and specific support seeking behaviors might combine to affect mental health. Working with undergraduate psychology students, for example, Starr (6) asked participants with internalizing symptoms to complete a daily diary of stressors, problem-oriented discussions, and mood for 2 weeks. They also completed trait-based measures of rumination, co-rumination, and excessive reassurance seeking. Individuals with high co-rumination scores showed elevated depressive symptoms specifically on days where they had engaged in more problem-oriented discussions.

Until recently, little research has examined co-rumination and associations with mental health in the context of online support seeking. While co-rumination can readily occur in face-to-face conversation, it is unclear whether online forums afford the same risks of rumination and to what degree. To investigate how common co-rumination is in online contexts, Battaglini et al. (28) used daily diary methodology to track co-ruminative tendencies in person, by phone, by text, and by social media. They found that young adolescents reported co-ruminating across all forms of communication, and that different forms had different psychosocial implications. Although face-to-face co-rumination was most common, co-rumination *via* text and social media was more common for girls than for boys. Further, while co-rumination *via* phone and in person increased positive affect and relational closeness, co-rumination *via* social media reduced positive affect. Similarly, Ohannessian et al. (29) found that co-rumination acted as a mediator in the relationship between social media use and anxiety, such that social media use predicted later co-rumination, which in turn predicted higher anxiety symptoms in early adolescents. Interestingly, however, other findings relating to online coping and co-rumination are equivocal. Following COVID-19, Stone and Veksler (9) showed that co-rumination about the pandemic and time on social media each predicted adults’ health anxiety, depressive symptoms, and state anxiety. Among adolescent girls, however, Van Zalk and Tillfors (30) showed that co-rumination with online friends decreased the influence of social anxiety on depressive symptomology. Further, while Frison et al. (31) found positive concurrent relationships between private Facebook interactions, online co-rumination, and depressive symptoms, these same private Facebook interactions predicted an increased perception of online social support 6 months later. Thus, while the frequency of online communication appears to be related to increased co-rumination, further research is needed to determine whether online support seeking as a coping strategy is related to co-rumination.

This study aimed to investigate co-rumination in the context of support seeking from close friends and in online contexts. Specifically, we examine whether co-rumination mediates the relationship between seeking support (from friends and online) and two indicators of mental health: depression and anxiety. Our first hypothesis was that seeking support from friends will be directly related to lower levels of depression and anxiety, which reflects the notion that seeking support from friends is an adaptive coping strategy (1). Second, and in line with previous findings (2, 3), we expected that online support seeking would be directly related to higher levels of depression and anxiety. Third, we hypothesized that seeking support from friends and online will be related to higher levels of co-rumination, as co-rumination is likely to occur in support seeking contexts (26, 29). We expected co-rumination to be related to higher levels of depression and anxiety (21, 24), thus highlighting a mediating relationship.

We specifically focus on adolescent girls for three reasons: (1) girls have a higher incidence of depression and anxiety (32–35), (2) girls are more likely to cope *via* support seeking, both in-person (36–40), and online (41, 42), and (3) girls are more likely to co-ruminate with same-sex friends (5, 21, 23). The phenomena of poorer mental health, higher support seeking, and higher co-rumination in adolescent girls suggests that girls are a potentially at-risk group that warrant focused research attention.

## Materials and methods

### Participants

The participants in this study were 186 girls (*M* = 13.64 years, *SD* = 1.03) from four independent schools in Sydney, Australia. Their ages ranged from 10.29 years to 15.33 years with 78 girls in Grade 7 (*M* = 12.55 years, *SD* = 0.46) and 108 girls in Grade 9 (*M* = 14.43 years, *SD* = 0.40). In each school, the proportion of students with language backgrounds other than English varied from 31% to 52%. These proportions are typical within the Sydney urban area, where 38.3% of households have a language background other than English (43). All schools were in the upper quartile on the Index of Community Socio-educational Advantage (ICSEA), suggesting that the student cohort was at a relative socioeconomic advantage when compared with other student cohorts in Australia (44). The ICSEA is a measure of a school’s educational advantage, incorporating student and school demographic factors.

### Measures

#### Seeking support for social stressors

Adolescents’ tendencies to seek support from friends and online were assessed *via* four vignettes. The vignettes depicted common social stressors (e.g., *You had a fight with your parents*; *You were excluded from an activity by your classmates*) and were adapted from the Motivational Theory of Coping Scale-12 (MTC-12) (45). The original MTC-12 incorporated video vignettes: in the current study these were converted to written narrative to support classroom-based survey administration. For each vignette, participants rated how likely they would be seek support from close friends and online on a scale from 1 (*not at all*) to 5 (*definitely*). For example, to measure online support seeking, participants were asked, “How much would you go online or text to talk to someone about it?” in response to each of the four vignettes. Vignettes were used this study to standardize the stressor contexts across participants and thus control the type of stressor considered in each participant’s response. Internal consistencies (Cronbach’s α; McDonald’s ω) for the subscales were α = 0.72; ω = 0.69 for seeking support from friends and α = 0.82, ω = 0.82 for online support seeking, demonstrating acceptable internal reliability.

#### Co-rumination

Co-rumination was measured using a short form (10) of the Co-rumination Questionnaire (5). This short form version of the Co-rumination Questionnaire includes one item for each of the nine components of co-rumination identified by Rose (5). The validity and reliability of the short form have been established in adolescent samples (10, 22), and the internal consistency for this sample was acceptable (α = 0.86; ω = 0.86).

#### Depression and anxiety

Depression and anxiety were measured using the depression and anxiety subscales of the Depression, Anxiety, and Stress Scale-Youth (DASS-Y) (46). The DASS-Y is a 21-item version of the DASS (47) that was adapted for use with children and adolescents in grades 3–12 (46). The depression and anxiety subscales of the DASS-Y consisted of seven items. Participants rated how much they had experienced each symptom in the preceding week on a 4-point Likert scale between 0 (*not true of you*) and 3 (*very true of you*). Higher scores indicated an increased severity of the emotional state being experienced in the past week. The validity of the DASS-Y has been established in a large adolescent sample (46) and internal consistencies for the current study were α = 0.90; ω = 0.91 for the depression subscale and α = 0.86; ω = 0.87 for the anxiety subscale.

### Procedure

Ethical approval was granted by the institutional ethics committee prior to the commencement of the study. All students in Grades 7 and 9 at the four participating schools were then invited to participate, with 21.20% of those in Grade 7 and 30.59% of those in Grade 9 agreeing. All participating students received written parental consent and students themselves provided written and verbal consent. The measures were then administered by the lead author during school hours *via* a paper-based survey conducted across Term 2 and 3, 2015. The survey protocol included a verbal reminder that the survey was about whom they turned to for help and advice, including using technology to ask for support from friends. Imputation of the subscale mean was used to estimate missing values for five students: two students missed a single coping item, and three students missed a DASS-Y item in their survey. Mean imputation is the recommended method of dealing with missing values in the DASS (47).

## Results

### Analysis strategy

Analyses were conducted using SPSS 25 and MPlus 8.0 (48). First, a series of congeneric measurement models were tested for each latent variable to be included in the structural model. A Confirmatory Factor Analysis (CFA; full measurement model) was then implemented to assess construct validity of the instruments and determine the relationships among latent variables. Finally, a structural equation model was tested to examine the direct and indirect effects of online support seeking and seeking support from friends on depression and anxiety, with co-rumination included as a hypothesized mediator. Model fit was evaluated using the Tucker-Lewis index (TLI), the comparative fit index (CFI), the root mean square error of approximation (RMSEA), and the standardized root mean square residual (SRMR). The following criteria were used to determine model fit: TLI and CFI greater than 0.95, RMSEA less than 0.05, and SRMR less than 0.08 (49). χ^2^ is reported but was not used as an indicator of model fit due to sensitivity to smaller sample sizes (50). The latent factors were not normally distributed, and a Satorra–Bentler χ^2^ *post-hoc* adjustment was used to account for this in the relevant models [maximum likelihood parameter estimates with standard errors (MLM) estimator].

### Measurement models

Development of the final scales included the testing of one-factor congeneric measurement models for each of the latent variables: seeking support from friends, online support seeking, co-rumination, depression, and anxiety. None of these models fitted the data well, and an examination of face validity and modification indices were used to determine items to be removed to improve measurement validity based on model fit. For example, in the original seeking support from friends and online support seeking scales, the vignette “You see your parents having a fight” had the highest modification indices. This vignette was also substantially different from the other vignettes, which depicted the adolescent being involved in the hypothetical stressor. A rationale for the removal of each item in all latent factors is provided in Supplementary Table 1. This process resulted in the retention of three items in each of the seeking support from friends and online support seeking factors, six items in each of the co-rumination and anxiety factors, and five items in the depression factor. Items that were retained and their factor loadings are included in Supplementary Table 2.

Following the item selection, a CFA was conducted in which all the retained items and their latent factor loadings were included in a single model (a full measurement model) to confirm the factor structure of the latent factors specified in the structural model and determine the relationships between latent factors. This model provided acceptable fit to the data (RMSEA = 0.05, SRMR = 0.07, TLI = 0.92, CFI = 0.93, SB-χ^2^ = 323.12, *df* = 220, *p* = 0.00). Factor loadings and reliabilities are shown in Table 1 and correlations between latent factors and descriptive statistics are shown in Table 2. Online support seeking was positively related to seeking support from friends, co-rumination, depression, and anxiety. Co-rumination was related to seeking support from friends, but not related to depression or anxiety. Depression and anxiety were also strongly correlated with one another.

**TABLE 1:** Factor loadings for each latent factors and scale reliabilities.

	Seeking support from friends	Online support seeking	Co-rumination	Depression	Anxiety
**Items**	**Factor loadings**				
SSF1	0.68	–	–	–	–
SSF2	0.77	–	–	–	–
SSF3	0.48	–	–	–	–
OSS1	–	0.76	–	–	–
OSS2	–	0.77	–	–	–
OSS3	–	0.71	–	–	–
CR1	–	–	0.60	–	–
CR2	–	–	0.62	–	–
CR3	–	–	0.71	–	–
CR4	–	–	0.76	–	–
CR5	–	–	0.73	–	–
CR6	–	–	0.65	–	–
DEP1	–	–	–	0.84	–
DEP2	–	–	–	0.52	–
DEP3	–	–	–	0.84	–
DEP4	–	–	–	0.79	–
DEP5	–	–	–	0.81	–
ANX1	–	–	–	–	0.71
ANX2	–	–	–	–	0.68
ANX3	–	–	–	–	0.75
ANX4	–	–	–	–	0.57
ANX5	–	–	–	–	0.61
ANX6	–	–	–	–	0.80
**Scale reliabilities**					
Cronbach’s α	0.65	0.79	0.83	0.87	0.84
McDonald’s ω	0.66	0.79	0.83	0.89	0.85

SSF, seeking support from friends; OSS, online support seeking; CR, co-rumination; DEP, depression; ANX, anxiety.

**TABLE 2:** Correlations between latent factors and descriptive statistics.

	Seeking support from friends	Online support seeking	Co-rumination	Depression	Anxiety
**Latent factor correlations**					
Seeking support from friends	–				
Online support seeking	0.28***	–			
Co-rumination	0.44***	22**	–		
Depression	−0.23**	0.21*	0.07	–	
Anxiety	−0.12	0.20*	0.10	0.76***	–
**Descriptive statistics**					
Mean (*SD*)	3.57 (0.98)	1.82 (0.94)	2.91 (0.91)	2.62 (3.53)	3.58 (4.10)
Range (min–max)	4.00 (1.00–5.00)	4.00 (1.00–5.00)	3.83 (1.17–5.00)	15.00 (0–15.00)	16.00 (0–16.00)
Skewness (*SE*)	−0.56 (0.18)	1.20 (0.18)	0.28 (0.18)	1.61 (0.18)	1.43 (0.18)
Kurtosis (*SE*)	−0.16 (0.36)	1.00 (0.36)	−0.53 (0.36)	1.90 (0.36)	1.43 (0.36)

*Correlation is significant at the 0.05 level (2-tailed); **Correlation is significant at the 0.01 level (2-tailed); ***Correlation is significant at the 0.001 level; *SD*, standard deviation. Latent factor scores are corrected for reliability.

### Structural model

**FIGURE 1:**
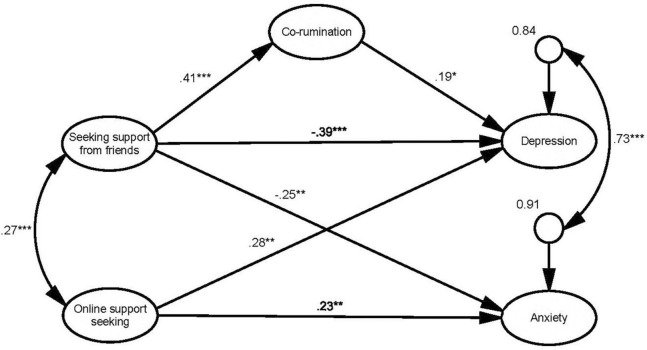
Structural model with standardized path coefficients (significant paths only). Indicators and uniquenesses are omitted for clarity; *significant at the 0.05 level (2-tailed); **significant at the 0.01 level (2-tailed); ***significant at the 0.001 level.

The hypothesized structural model (Figure 1), in which co-rumination mediated the relationship between support seeking and mental health, was a good fit for the data (RMSEA = 0.05, SRMR = 0.07, TLI = 0.92, CFI = 0.93, SB-χ^2^ = 323.12, *df* = 220, *p* = 0.00). Given that both the measurement and the fully forward structural model had the same degrees of freedom, the model fit indices were identical.

There were direct negative relationships between seeking support from friends and both depression (β = −0.39, *p* < 0.001) and anxiety (β = −0.25, *p* = 0.01), suggesting that girls who indicated a stronger likelihood of seeking support from their friends for social stressors were less likely to experience poor mental health. There was also indication that co-rumination mediated the relationship for depression. Specifically, we found an inconsistent mediation (suppression) effect (51), as seeking support from friends was positively related to co-rumination (β = 0.41, *p* < 0.001), which in turn was positively related to depression (β = 0.19, *p* = 0.04). Thus, the indirect effect of seeking support from friends on depression *via* co-rumination was 0.08, and the total effect was −0.31. Co-rumination therefore appears to reduce the beneficial effect of seeking support from friends on depression. Co-rumination was not related to anxiety, however.

Online support seeking was positively related to both depression (β = 0.28, *p* < 0.001) and anxiety (β = 0.23, *p* = 0.01) but was not related to co-rumination. Thus, girls who identified that they were more likely to seek support online were more likely to experience depression and anxiety but were not more likely to co-ruminate with their friends.

## Discussion

This study examined the potential mediating role of co-rumination in the relationship between support seeking and mental health (depression and anxiety) in adolescent girls. It extended previous work by considering support seeking from friends in traditional contexts and in online contexts. As hypothesized, co-rumination mediated the relationship between seeking support from friends and depression. In the absence of co-rumination, there was a lower frequency of mental health concerns for girls seeking support from friends. Support-seeking from friends was also likely to predict co-rumination, however, which in turn predicted higher depression. Interestingly, co-rumination did not mediate the relationship between online support seeking and either anxiety or depression. Online support seeking was instead directly related to higher levels of depression and anxiety, but unrelated to co-rumination.

### Support seeking from friends and mental health

The first key finding of this study was that in the absence of co-rumination, seeking support from friends for social stressors was related to lower levels of depression and anxiety. This aligns with some, but not all, previous research (12–14) and supports the notion of support seeking from friends as an adaptive coping strategy for adolescents in response to everyday stressors. One of the novel contributions of this study was the finding that co-rumination suppressed the beneficial effect of seeking support from friends and depression: adolescents who identified they would seek support from their friends reported higher levels of co-rumination, which was in turn was related to higher depression scores. The positive relationship between seeking support from friends and co-rumination is unsurprising, given the extensive body of evidence that connects co-rumination with increases in friendship quality [e.g., (7, 8)]. Our findings also align with and extend those of Vélez et al. (15), who reported that support seeking was a useful coping strategy for adolescents if they also engaged in low levels of rumination, but that support seeking became problematic in the presence of high levels of rumination. Thus, the specific strategies that adolescents use while seeking support from friends appear to influence the effectiveness of support seeking, such that co-rumination “may lead social support attempts to backfire, contributing to (rather than protecting against) depressed mood” [(6), p. 9]. As friends are only one of several sources of support that adolescents may turn to, we recommend that future research examines both the extent and effect of co-rumination with other support providers (e.g., parents, siblings, other family members) on adolescent mental health.

### Online support seeking and mental health

We also found that seeking support online for social stressors was related to higher levels of both depression and anxiety. This contributes to a growing body of literature that suggests that informal online support seeking is a problematic way of coping with stress for adolescents, given its direct relationship with negative mental health indicators (2–4). While not investigated in this study, previous research offers several potential reasons for why seeking support online represents a less adaptive way of coping with stress. The quality of support received is a subjective evaluation of the support seeker (12), and adolescents in qualitative studies have reported that reduced social cues in online, text-based contexts can limit the quality of support and emotional relief that can be provided in response to support seeking attempts (16, 17). These reduced social cues can also contribute to hostility, flaming, and other negative responses (52), which could feasibly exacerbate the stress that generated the support seeking attempt. Given that many adolescents do report seeking informal support online [e.g., (2, 3)], we recommend further research into the reasons why online support seeking is related to poorer mental health and strategies to encourage adolescents to choose more useful modes of seeking informal support.

### Online support seeking and co-rumination

While previous studies have demonstrated that co-rumination occurs in online contexts (28, 29), ours is the first to our knowledge to examine the relationship between online support seeking and co-rumination specifically. Contrary to our hypothesis, online support seeking was not related to co-rumination in our model. Thus, while online support seeking and co-rumination were positively correlated, when controlling for seeking support from friends this relationship was not significant. This suggests that seeking support from friends in person is more conducive to engaging in co-rumination, which is supported by previous findings (28). We speculate that there are several possible reasons why co-ruminative conversations might be constrained in online contexts. For example, in comparison to engaging in face-to-face conversations, there is relatively more effort that is required to type supportive messages and conversations may occur asynchronously. Given the repetitive and ongoing nature of co-rumination, these characteristics of online, text-based communication may make co-rumination more difficult than in-person. In addition, when interacting online, adolescents are likely to be managing multiple conversations with peers simultaneously (20), which could disrupt the flow of co-ruminative conversations.

It should be noted that we used a general measure of co-rumination that did not specify whether co-rumination took place in online or face-to-face contexts. Previous research suggests that social media use is related to increases in *online* co-rumination (29, 31): our study found that the intention to seek support online was not related to increased general co-rumination. This could indicate that frequent social media use is more likely to lead to co-rumination rather than a preference for seeking support online. Alternatively, online support seeking may be related to increased *online* co-rumination rather than general co-rumination. We recommend that future research focusing on online support seeking includes measures of online co-rumination and frequency of social media use to explore the nature of this relationship.

## Limitations and conclusion

There are several limitations to note. First, the cross-sectional design of this study prevents conclusions being drawn regarding the direction of relationships between study variables. For example, while we found that co-rumination and online support seeking were concurrently associated with experiencing higher levels of depression and anxiety, it is possible that adolescent girls who are already suffering poor mental health are more likely to use problematic online interpersonal coping mechanisms, including co-rumination (31, 53). While longitudinal studies are needed to determine the direction of effects, we suggest that bidirectional relationships between support seeking strategies and mental health are likely. Such longitudinal studies would also allow for investigation of potential developmental differences, which were beyond the scope of this study. Second, the generalizability of our findings is limited as our sample consisted of girls from socioeconomically advantaged school contexts. While we had a specific interest in this population due to their increased risk of mental health difficulties (54–56), further research is needed in male and more socioeconomically diverse populations. Third, data collection also took place in school time while participants were with their peers. It is therefore plausible that girls’ responses were influence by them being near their peers, however, the likelihood of this was minimized as the survey was completed under supervision. Finally, we used hypothetical vignettes of social stressors to create standardized stressors against which all participants rated their likelihood of seeking support from friends and online. While the use of vignettes is a common and established methodology that allows differences in the adolescents’ exposure to each stressor type to be controlled (57, 58), a limitation of this measurement approach is that intended support seeking behaviors may be different to actual behaviors (59). However, given the relationships between adolescents’ support seeking intentions and depression and anxiety, we suggest that both intentions and actual behaviors are significant.

Despite these limitations, the current study makes an important contribution to our understanding of the effect of co-rumination on the relationship between support seeking and depression. This study also contributes to a growing body of evidence that suggests that online support seeking is related to higher levels of depression and anxiety. These findings may be used to guide intervention efforts that aim to assist adolescents in developing adaptive interpersonal coping strategies. One important implication of these findings, for example, is that parents, teachers, and clinicians should be wary of encouraging adolescents to discuss problems online. Further, while seeking support from friends appears to be an adaptive coping strategy, there may also be negative implications for mental health if this involves repetitive discussion of problems. A final implication is that clinicians should be aware of, and address adolescents’ potential use of co-rumination and online support seeking as a way of coping with everyday stressors, given their direct relationships with poorer mental health. For example, during assessment and/or case review, clinicians would do well to provide psychoeducation about the possible negative effects of online support seeking and co-rumination and divert clients to more positive coping strategies. Future research should extend these findings by examining the long-term implications of online support seeking for adolescent mental health, and by confirming the generalizability of these findings for other populations.

## Data availability statement

The datasets presented in this article are not readily available because this dataset is not available due to ethics protocols. Requests to access the datasets should be directed to EM, e.mackenzie@westernsydney.edu.au.

## Ethics statement

The studies involving human participants were reviewed and approved by Macquarie University. Written informed consent to participate in this study was provided by the participants’ legal guardian/next of kin.

## Author contributions

EM, AM, and PV conceptualized the study. EM collected the data. EM and RP conducted the analyses. All authors drafted and reviewed the manuscript and approved the final submitted version.
