# Supplementary material for: Online support seeking, co-rumination, and mental health in adolescent girls
Source: Front Psychiatry. 2023 Mar 7;14:1040636. doi: 10.3389/fpsyt.2023.1040636 (PMC10027699; doi:10.3389/fpsyt.2023.1040636)
Supplement: Supplementary file 1 [file Table_1.docx]

Supplementary Material

Online support seeking, co-rumination, and mental health in adolescent girls

Erin Mackenzie^*^, Anne McMaugh, Penny Van Bergen, and Roberto H. Parada

*** Correspondence:** Corresponding Author: [e.mackenzie@westernsydney.edu.au](mailto:e.mackenzie@westernsydney.edu.au)

# Supplementary Tables

Supplementary Table 1

*Rationale for removal of items from original measures during model building*

| Latent factor | Item | Retained in model? | Rationale for item removal |
| --- | --- | --- | --- |
| Seeking support from friends  (adapted from Zimmer-Gembeck et al., 2012) | SSF1: You are being bullied at school. How much would you go and seek the support or help of a close friend? | Yes | - |
|  | SSF2: You were excluded from an activity by your classmates. How much would you go and seek the support or help of a close friend? | Yes | - |
|  | SSF3: You had a fight with your parents. How much would you go and seek the support or help of a close friend? | Yes | - |
|  | SSF4: You see your parents having a fight. How much would you go and seek the support or help of a close friend? | No | Highest MI (36.02 with SSF3); poor face validity as other items depict adolescent as being involved in the social stressor. |
| Online support seeking  (adapted from Zimmer-Gembeck et al., 2012) | OSS1: You are being bullied at school. How much would you go online or text to talk to someone about it? | Yes | - |
|  | OSS2: You were excluded from an activity by your classmates. How much would you go online or text to talk to someone about it? | Yes | - |
|  | OSS3: You had a fight with your parents. How much would you go online or text to talk to someone about it? | Yes | - |
|  | OSS4: You see your parents having a fight. How much would you go online or text to talk to someone about it? | No | Highest MI (10.71 with OSS3); poor face validity as other items depict adolescent as being involved in the social stressor. |
| Co-rumination  (items from Rose, 2002) | CR1: When we see each other, if one of us has a problem, we will talk about the problem even if we had planned to do something else together. | Yes | - |
|  | CR2: When I have a problem, my friend always tries to get me to tell every detail about what happened. | Yes | - |
|  | CR3: When we talk about a problem that one of us has, we talk about all of the reasons why the problem might have happened. | Yes | - |
|  | CR4: When we talk about a problem that one of us has, we try to figure out every one of the bad things that might happen because of the problem. | Yes | - |
|  | CR5: When we talk about a problem that one of us has, we spend a lot of time trying to figure out parts of the problem that we can’t understand. | Yes | - |
|  | CR6: When we talk about a problem that one of us has, we talk a lot about how bad the person with the problem feels. | Yes | - |
|  | CR7: We talk about problems that my friend or I are having almost every time we see each other. | No | Highest MI in initial CFA for 9-item co-rumination scale (18.16 with CR1). Inspection of face validity shows item measures frequency of problem talk rather than focus of co-ruminative discussions. |
|  | CR8: When my friend has a problem, I always try really hard to keep my friend talking about it. | No | Highest MI in third CFA for 7-item co-rumination scale (9.60 with CR4; 9.00 with CR2). Inspection of face validity shows item measures frequency of problem talk rather than focus of co-ruminative discussions. |
|  | CR9: When we talk about a problem that one of us has, we’ll talk about every part of the problem over and over. | No | Highest MI in second CFA for 8-item co-rumination scale (18.45 with CR2). Inspection of face validity shows item measures frequency of problem talk rather than focus of co-ruminative discussions. |
| Depression (items from Szabo & Lovibond, 2022) | DEP1: I could not stop feeling sad. | Yes | - |
|  | DEP2: There was nothing nice I could look forward to. | Yes | - |
|  | DEP3: I hated myself. | Yes | - |
|  | DEP4: I felt like I was no good. | Yes | - |
|  | DEP5: I felt that life was terrible. | Yes | - |
|  | DEP6: I did not enjoy anything. | No | Highest MI in initial CFA for 7-item depression scale (17.49 with DEP2). DEP6 also had a high MI with DEP3 (14.74). As DEP2 and DEP6 both measured a lack of enjoyment in activities, the removal of DEP6 enabled this aspect of depression to be measured in the final model. |
|  | DEP7: I hated my life. | No | Highest MI in second CFA for 6-item depression scale (9.19 with DEP1). As both DEP5 and DEP7 measured the “devaluation of life” component of depression (Szabo and Lovibond 2022), DEP7 was removed as this component would still be represented in the final model by DEP5. |
| Anxiety (items from Szabo & Lovibond, 2022) | ANX1: My hands felt shaky. | Yes | - |
|  | ANX2: I could feel my heart beating really fast, even when I was not exercising. | Yes | - |
|  | ANX3: I felt like I was about to panic. | Yes | - |
|  | ANX4: I had trouble breathing (e.g. fast breathing), even when I wasn't exercising and I was not sick. | Yes | - |
|  | ANX5: I was dizzy, like I was about to faint. | Yes | - |
|  | ANX6: I felt scared for no good reason. | Yes | - |
|  | ANX7: I felt terrified. | No | Highest MI in initial CFA for 7-item anxiety scale (18.15 with ANX3). ANX7 also had a high MI with ANX5 (8.56). As ANX3, ANX5 and ANX7 all reflected the “subjective experience of anxious affect” (Szabo and Lovibond 2022), the removal of ANX7 enabled this aspect of anxiety to be measured by ANX3 and ANX5. |

*Note.* SSF = seeking support from friends; OSS = online support seeking; CR = co-rumination; DEP = depression; ANX = anxiety; MI = modification index; CFA = confirmatory factor analysis

Supplementary Table 2

*Factor Loadings of Items in Each Latent Factor*

| Latent factor | Item | Factor Loading |
| --- | --- | --- |
|  |  |  |
| Seeking support from friends  (adapted from Zimmer-Gembeck et al., 2012) | SSF1: You are being bullied at school. How much would you go and seek the support or help of a close friend? | .68 |
|  | SSF2: You were excluded from an activity by your classmates. How much would you go and seek the support or help of a close friend? | .77 |
|  | SSF3: You had a fight with your parents. How much would you go and seek the support or help of a close friend? | .48 |
| Online support seeking  (adapted from Zimmer-Gembeck et al., 2012) | OSS1: You are being bullied at school. How much would you go online or text to talk to someone about it? | .76 |
|  | OSS2: You were excluded from an activity by your classmates. How much would you go online or text to talk to someone about it? | .77 |
|  | OSS3: You had a fight with your parents. How much would you go online or text to talk to someone about it? | .71 |
| Co-rumination  (items from Rose, 2002) | CR1: When we see each other, if one of us has a problem, we will talk about the problem even if we had planned to do something else together. | .60 |
|  | CR2: When I have a problem, my friend always tries to get me to tell every detail about what happened. | .62 |
|  | CR3: When we talk about a problem that one of us has, we talk about all of the reasons why the problem might have happened. | .71 |
|  | CR4: When we talk about a problem that one of us has, we try to figure out every one of the bad things that might happen because of the problem. | .76 |
|  | CR5: When we talk about a problem that one of us has, we spend a lot of time trying to figure out parts of the problem that we can’t understand. | .73 |
|  | CR6: When we talk about a problem that one of us has, we talk a lot about how bad the person with the problem feels. | .65 |
| Depression  (items from Szabo & Lovibond, 2022) | DEP1: I could not stop feeling sad. | .84 |
|  | DEP2: There was nothing nice I could look forward to. | .52 |
|  | DEP3: I hated myself. | .84 |
|  | DEP4: I felt like I was no good. | .79 |
|  | DEP5: I felt that life was terrible. | .81 |
| Anxiety  (items from Szabo & Lovibond, 2022) | ANX1: My hands felt shaky. | .71 |
|  | ANX2: I could feel my heart beating really fast, even when I was not exercising. | .68 |
|  | ANX3: I felt like I was about to panic. | .75 |
|  | ANX4: I had trouble breathing (e.g. fast breathing), even when I wasn't exercising and I was not sick. | .58 |
|  | ANX5: I was dizzy, like I was about to faint. | .62 |
|  | ANX6: I felt scared for no good reason. | .80 |

*Note.* SSF = seeking support from friends; OSS = online support seeking; CR = co-rumination; DEP = depression; ANX = anxiety

**References**

Rose, A. J. (2002). Co–rumination in the friendships of girls and boys. *Child Development, 73*(6), 1830-1843.

Szabo, M., & Lovibond, P. F. (2022). Development and psychometric properties of the DASS-youth (DASS-Y): an extension of the depression anxiety stress scales (DASS) to adolescents and children. *Frontiers in Psychology*, 1547.

Zimmer-Gembeck, M. J., Skinner, E. A., Morris, H., & Thomas, R. (2013). Anticipated coping with interpersonal stressors: Links with the emotional reactions of sadness, anger, and fear. *The Journal of Early Adolescence, 33*(5), 684–709.
